# Disruption of the Fibroinflammatory Loop: A Therapeutic Strategy for Cardiac Fibrosis in Non-Ischemic Cardiomyopathy

**DOI:** 10.31083/RCM48589

**Published:** 2026-03-16

**Authors:** Hongyu Qiu, Inna P. Gladysheva

**Affiliations:** ^1^Translational Cardiovascular Research Center, Department of Internal Medicine, College of Medicine-Phoenix, University of Arizona, Phoenix, AZ 85004, USA; ^2^Clinical Translational Sciences (CTS) and Bio5 Institution, University of Arizona, Phoenix, AZ 85004, USA

**Keywords:** fibrosis, heart, inflammation, fibroblast, macrophage, cardiomyocyte, inflammasome

## Abstract

In non-ischemic cardiomyopathy, inflammation is closely associated with cardiac fibrosis, which significantly contributes to adverse outcomes and promotes heart failure (HF). Recent mechanistic studies have demonstrated that interactions between fibrotic and inflammatory pathways create a dynamic, self-perpetuating fibroinflammatory loop, thereby accelerating disease progression. New mono or combination therapies that target this cycle by blocking specific inflammatory signals, modulating the immune response, and altering extracellular matrix (ECM) stiffness may halt or even reverse fibrosis. This opinion article discusses critical recent discoveries, current obstacles, and future opportunities in developing inflammation-focused treatments for cardiac fibrosis in non-ischemic cardiomyopathies.

## 1. Introduction

Cardiac fibrosis, the excessive deposition of extracellular matrix (ECM) in the interstitium primarily by activated cardiac fibroblasts, directly contributes to both ischemic (caused by blocked arteries or myocardial infarction) and non-ischemic (caused by genetic factors, amyloidosis, aging, viral infections, diabetic or hepatitis disorders, and exposure to toxins or drugs) cardiac remodeling, the development of related cardiomyopathies, and their progression to symptomatic heart failure (HF). Historically, cardiac fibrosis was viewed as a passive response to injury or stress. However, accumulating evidence redefines it as a chronic inflammatory disease [1, 2, 3, 4], perpetuated by reciprocal signaling between immune cells, fibroblasts (primary source of myofibroblasts in adult hearts) [5], cardiomyocytes [6, 7], and the ECM [8, 9, 10, 11, 12, 13]. Understanding the mechanisms that drive this dynamic fibroinflammatory loop and accelerate disease progression is essential for developing therapies that target the underlying pathogenic processes and break the vicious cycle.

Although inflammation has been recognized as a key driver of cardiac fibrosis for decades, advances in single-cell and multi-omics profiling over the past few years have reshaped our understanding of how inflammation drives pathological fibrosis. Particularly, recent work paints a far more dynamic picture, e.g., how fibrosis, in turn, sustains inflammation. Immune cells and fibroblasts form a reciprocal, context-dependent signaling niche whose behavior determines whether the injured heart heals or progresses to maladaptive remodeling. These insights provide a rationale for therapies, whether mono- or combined, that target specific inflammatory pathways rather than broadly suppressing immunity.

This opinion summarizes recent advances in understanding the interplay between interstitial cardiac fibrosis and inflammation, highlighting mechanistic insights, translational strategies, and emerging therapies targeting the fibroinflammatory loop [14, 15, 16], focusing on non-ischemic cardiomyopathies and providing timely and actionable perspectives on future directions.

## 2. Inflammatory Crosstalk Driving Cardiac Fibrosis

Chronic inflammation promotes maladaptive cardiac dysfunction and cardiac fibrosis by releasing pro-inflammatory cytokines that damage cardiac cells and activating profibrotic pathways that lead to excessive remodeling and stiffening of the heart muscle.

### 2.1 Heterogeneity of Macrophage–Fibroblast Communication

Macrophages are the most abundant immune cells in the heart, derived from both resident and infiltrating lineages, which, in non-ischemic cardiomyopathies, drive cardiac fibrosis by both promoting and inhibiting it [17]. Recent single-cell studies have revealed substantial macrophage heterogeneity in the heart. It has been shown that resident macrophages and infiltrating monocyte-derived macrophages exhibit distinct gene expression profiles and phenotypes, and that their interactions with fibroblasts play divergent roles in cardiac fibrosis [18, 19, 20]. The recruited macrophages that highly express chemokine C-C motif receptor 2 (CCR2) promote fibroblast activation by secreting pro-inflammatory cytokines, e.g., interleukin-1 beta (IL-1β), tumor necrosis factor alpha (TNF-α), profibrotic transforming growth factor beta (TGF-β) [21], and matrix metalloproteinases (MMPs), facilitating fibroblast-to-myofibroblast transition and remodeling cardiac ECM [18, 22]. The resident CCR-negative (CCR2⁻) macrophage populations generally support homeostasis and tissue repair, balancing inflammation and limiting excessive fibrosis. The overall outcome depends heavily on timing and disease stage. This dualism underscores the need for therapeutic strategies that modulate macrophage phenotype rather than relying on broad depletion. The net result depends on timing and disease stages [12, 23].

### 2.2 Immune-Metabolic Reprogramming Toward Pro-Fibrotic Phenotypes

Emerging mechanistic studies identify immune-metabolic pathways as central mediators of fibrosis through immune–stromal crosstalk. Chronic inflammation reprograms macrophage and fibroblast metabolism toward glycolysis and pro-fibrotic phenotypes. Meanwhile, cytokine modulators, including Interleukin-11 (IL-11), have also emerged as potent pro-fibrotic mediators linking inflammatory stress to fibroblast activation. Experimental modulation of IL-11 signaling alters fibroblast phenotypes and fibrotic outcomes in preclinical models, suggesting that targeting IL-11 or downstream effectors could reduce pathological ECM deposition. However, clinical translation will require careful context-dependent evaluation [24, 25, 26].

### 2.3 Inflammasome Activation as a Pro-Fibrotic Sensor

Growing evidence identifies NOD-like receptor family pyrin domain-containing 3 (NLRP3) (NACHT, Leucine-Rich Repeat (LRR) and Pyrin Domain (PYD) domains-containing protein 3) as a key mediator of pathological fibrosis. NLRP3 is a key component of the inflammasome, a multi-protein complex that activates inflammatory responses in cells. Activation of NLRP3 in cardiac cells, including fibroblasts and resident immune cells, leads to caspase-1 activation, release of IL-1β and IL-18, and pyroptotic signaling, establishing a feed-forward loop that drives fibroblast activation and persistent inflammation. Inhibiting this pathway in preclinical models reduces fibroblast activation and limits ECM deposition, suggesting that the inflammasome is not merely a bystander, but a central orchestrator of fibrotic remodeling [27, 28, 29, 30].

### 2.4 Pro-Fibrotic Neurohormonal Activation

Chronic inflammation activates the renin-angiotensin-aldosterone system (RAAS), leading to sodium and water retention, vasoconstriction, and increased blood pressure. It also impairs the natriuretic peptide system, which is designed to counteract the harmful effects of persistent RAAS activation [1, 31, 32, 33]. As a result, the combined pathological dysregulation of these systems leads to neurohormonal activation that promotes cardiac fibrosis by directly stimulating pro-fibrotic pathways, including the TGF-β/Smad (small mothers against decapentaplegic) [21], which are activated by angiotensin II (Ang II), while the anti-fibrotic effects of the corin-atrial natriuretic peptide (ANP) axis are impaired [32, 34, 35].

## 3. Fibrosis Acts as an Inflammatory Amplifier

Beyond being a downstream consequence of inflammation, fibrotic ECM actively feeds back to amplify the inflammatory process. The stiffened matrix stores pro-inflammatory mediators and generates mechanical cues that enhance inflammatory signaling. Increases in matrix stiffness activate mechanosensitive pathways in fibroblasts and immune cells, including YAP/TAZ (Yes-associated protein/Transcriptional coactivator with PDZ-binding motif), integrins, and NF-κB (nuclear factor kappa-light-chain-enhancer of activated B cells), thereby sustaining inflammatory gene expression [13, 36, 37]. Chronic cardiac remodeling, including fibrosis, in combination with chronic neurohormonal activation, induces a state of sterile chronic inflammation or the expression of inflammatory genes, including *TNF-α*, *IL-6*, *IL-1β*, and *myostatin* by cardiomyocytes and fibroblasts, contributing to cardiac and systemic inflammation, which fosters macrophage infiltration and, in turn, further promotes cardiac interstitial fibrosis and dysfunction [38, 39, 40].

This establishes a self-reinforcing cycle: chronic inflammation activates fibroblasts and stimulates neurohormonal activation → interstitial fibrosis increases stiffness → stiffness amplifies inflammation → further fibrosis. Recognizing this loop reframes the ECM not merely as a passive downstream target but as an active driver of disease progression [41]. This shift is redefining therapeutic goals that aim not only to reduce collagen deposition but also to disrupt the mechanochemical signaling loops that maintain chronic fibrosis.

## 4. Therapeutic Implications

Advances in mechanistic understanding are shifting therapeutic strategies from broad anti-inflammatory approaches toward targeted interruption of the interconnected crosstalk loop between inflammation and cardiac fibrosis, or toward combined interventions.

### 4.1 Precision Immunomodulation

Given the heterogeneity of cardiac macrophages, therapies that reprogram immune phenotypes may prove more effective than generalized immunosuppression. For example, modulating CCR2 signaling to limit recruitment of pro-fibrotic monocyte-derived macrophages, or promoting resident macrophage-like phenotypes with anti-inflammatory and pro-resolving functions.

Targeting metabolic reprogramming in macrophages aims to shift them away from glycolytic, pro-fibrotic states. Recent strategies also focus on neutralizing pro-fibrotic cytokines, such as IL-11, to reprogram both macrophage and fibroblast phenotypes. Although direct TGF-β inhibition reduces fibrosis in experimental models, systemic safety concerns limit its clinical use. Newer approaches emphasizing downstream or context-restricted blockade, such as targeting fibroblast-specific TGF-β effectors or IL-11 signaling, may preserve anti-fibrotic efficacy while reducing adverse effects [29]. In addition, approaches targeting the precise modulation of TNF-α, IL-6, IL-1β, and myostatin secreted by cardiomyocytes and fibroblasts could slow or reverse cardiac fibrotic remodeling and prevent macrophage infiltration. Successful clinical translation will require careful consideration of safety and patient stratification.

Given the central role of inflammasome activation, selective inhibitors or modulators of the NLRP3-containing inflammasome and its downstream cytokines (IL-1β, IL-18) may help blunt pathological inflammation and subsequent fibrosis [42, 43]. The key challenge is suppressing pathogenic signaling without impairing essential reparative responses.

### 4.2 Combining Intervention

Combining therapies that interrupt the mechanochemical feedback loop between fibrosis and inflammation by targeting distant components of this interdependent pathological circle may offer particular benefit and be superior to mono-therapies. Potential strategies include altering ECM stiffness or composition with ECM-modulating biologics, blocking mechano-transduction pathways—such as YAP/TAZ and integrins—in immune and stromal cells, or combining anti-fibrotic and anti-inflammatory treatments with drugs that normalize dysregulated neurohumoral systems.

### 4.3 Targeted Delivery and Combination Therapies

Advances in nanoparticle platforms, cell-targeting antibodies, and localized delivery approaches are enabling more precise therapeutic targeting, minimizing off-target effects. Because inflammation and fibrosis are tightly intertwined, combination regimens, such as selective anti-inflammatory agents paired with fibroblast modulators and hemodynamic therapy, are increasingly viewed as the most rational approach to achieving meaningful reversal of remodeling. Early preclinical studies suggest synergistic effects, though confirmation in human studies remains forthcoming [44].

### 4.4 Repurposed and Adjunctive Therapies

Recent preclinical and early translational work suggests that drugs with established cardiovascular indications may have anti-fibrotic and immunomodulatory effects. Drugs such as SGLT2 (sodium-glucose cotransporter 2) inhibitors and colchicine, initially developed for metabolic or gout indications, appear to have cardioprotective, partially anti-inflammatory effects and may modulate remodeling [45, 46, 47]. Emerging studies show these drugs may modulate profibrotic signaling, immune activation, and fibroblast biology beyond their metabolic effects [42]. While promising at the population level, such agents have not yet been proven to reverse established myocardial fibrosis, underscoring the need for targeted strategies in high-risk, inflammation-driven cohorts [10].

## 5. Challenges and Future Directions

Despite conceptual progress, several barriers persist. (1) Identifying patients with active inflammation-driven fibrosis remains challenging. (2) Timing is crucial: interventions that blunt inflammation too early may impair necessary repair; started too late, they may not reverse entrenched fibrosis. (3) Most promising approaches remain preclinical and require translational pipelines that incorporate human tissue platforms, refined biomarkers, and adaptive clinical trial designs.

Key priorities moving forward include: (1) validating circulating and imaging biomarkers linked to pathogenic macrophage–fibroblast-cardiomyocyte states; (2) advancing fibroblast-, cardiomyocyte- and macrophage-targeted biologics with safety-focused strategies; (3) developing combination regimens guided by mechanistic biomarkers; and (4) leveraging translationally-relative animal models, human organoid and engineered tissue platforms for target validation and safety screening.

## 6. Conclusion

**Fig. 1.**
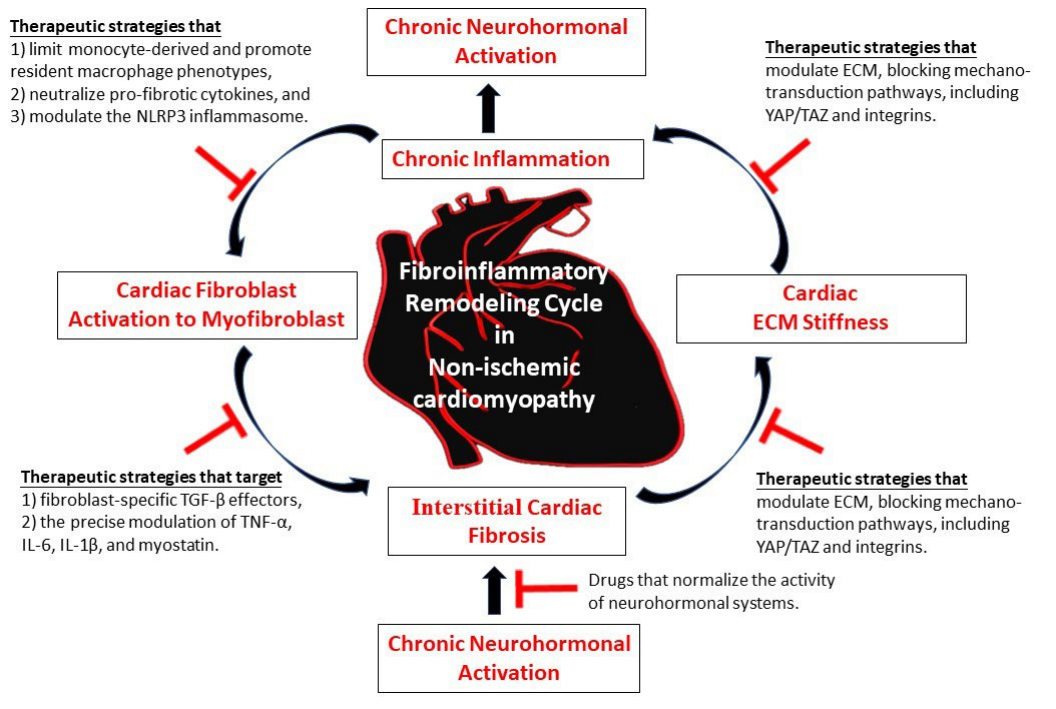
**Simplified schematic presentation of the fibroinflammatory remodeling in non-ischemic cardiomyopathy as a self-reinforcing cycle that promotes cardiac interstitial fibrosis**. Targeted mono or combined interruption of the interlinked loop between chronic inflammation and interstitial cardiac fibrosis may represent a promising anti-fibrotic therapeutic strategy.

As schematically illustrated in Fig. 1, recent advances in research have reshaped the understanding of cardiac fibrosis as a dynamic, inflammatory process driven by interactions among immune cells, fibroblasts, cardiomyocytes, and the ECM. The growing mechanistic evidence warrants a therapeutic shift toward targeted interventions that normalize communication among cardiac immune, stromal, and muscular cells while disrupting the mechanical feedback loops that drive chronic fibroinflammatory remodeling. Although adopting this new paradigm is challenging, it may be essential for developing therapies that not only slow fibrosis progression but also reverse it, thereby restoring a healthier and more resilient myocardium. Strategies that target the immune–fibroblast–cardiomyocyte-ECM crosstalk show promise for stopping or reversing remodeling. Combining mechanistic insights with translational tools and biomarker-guided approaches will be crucial for creating effective clinical treatments.
